# Implementation of uniform information on fetal movement in a Norwegian population reduced delayed reporting of decreased fetal movement and stillbirths in primiparous women - a clinical quality improvement

**DOI:** 10.1186/1756-0500-3-2

**Published:** 2010-01-04

**Authors:** Eli Saastad, Julie Victoria Holm Tveit, Vicki Flenady, Babill Stray-Pedersen, Ruth C Fretts, Per E Børdahl, J Frederik Frøen

**Affiliations:** 1 Norwegian Institute of Public Health, Division of Epidemiology, Oslo, Norway; 2 Akershus University College, Lillestrøm, Norway; 3 Department of Obstetrics and Gynecology, Centre for Perinatal Research, Rikshospitalet University Hospital, Oslo, Norway; 4 University of Oslo, Medical faculty, Norway; 5 Department of Obstetrics and Gynecology, University of Queensland, Mater Mothers' Hospital, South Brisbane, Australia; 6 Brigham and Women's Hospital, Division of Maternal-Fetal Medicine, Harvard Medical School, Boston, MA and Harvard Medical Associates, Wellesley, MA, USA; 7 Institutes for Clinical Medicine, Section for Gynecology and Obstetrics, and University of Bergen, Norway

## Abstract

**Background:**

Delayed maternal reporting of decreased fetal movement (DFM) is associated with adverse pregnancy outcomes. Inconsistent information on fetal activity to women during the antenatal period may result in delayed reporting of DFM. We aimed to evaluate an intervention of implementation of uniform information on fetal activity to women during the antenatal period.

**Methods:**

In a prospective before-and-after study, singleton women presenting DFM in the third trimester across 14 hospitals in Norway were registered. Outcome measures were maternal behavior regarding reporting of DFM, concerns and stillbirth. In addition, cross-sectional studies of all women giving birth were undertaken to assess maternal concerns about fetal activity, and population-based data were obtained from the Medical Birth Registry Norway.

**Results:**

Pre- and post-intervention cohorts included 19 407 and 46 143 births with 1 215 and 3 038 women with DFM respectively. Among primiparous women with DFM, a reduction in delayed reporting of DFM (≥48 hrs) OR 0.61 (95% CI 0.47-0.81) and stillbirths OR 0.36 (95% CI 0.19-0.69) was shown in the post-intervention period. No difference was shown in rates of consultations for DFM or maternal concerns. Stillbirth rates and maternal behavior among women who were of non-Western origin, smokers, overweight or >34 years old were unchanged.

**Conclusions:**

Uniform information on fetal activity provided to pregnant women was associated with a reduction in the number of primiparous women who delayed reporting of DFM and a reduction of the stillbirth rates for primiparous women reporting DFM. The information did not appear to increase maternal concerns or rate of consultation. Due to different imperfections in different clinical settings, further studies in other populations replicating these findings are required.

## Background

Women presenting with decreased fetal movement (DFM) are at increased risk of fetal growth restriction, stillbirth, preterm birth and emergency caesarean section [1-5]. Excessive delay in maternal reporting of DFM is associated with perinatal deaths [5,6]. There is no agreement on any quantitative limit between "normal" versus "abnormal" fetal activity [7,8], due to normal variation among healthy fetuses [9] and variation in maternal ability to perceive fetal activity [10]. The only definition of DFM based on focused counting data in a total population, is the rule of "10 fetal movements within two hours", which subsequently has been tested as a screening tool [7,11]. Fetal movement counting (FMC) is a method used by the mother to quantify her baby's movements. Various methods with different alarm limits have been published; discussed elsewhere [7,8]. FMC is not promoted as a universal screening tool for fetal wellbeing [4], but has been recommended in high-risk pregnancies [12,13].

The most important clinical screening tool for DFM for identifying high-risk pregnancies is the women's own perception of a decrease [8,14-16]; i.e. her perception of a *change*, not the crossing of a given limit. Existing guidelines for antenatal care in the United Kingdom, the US and Norway recommend that a distinct reduction of fetal movement should be reported and lead to further investigation [17-20]. In our Norwegian setting nearly 100% of all pregnant women attend the public antenatal care program provided by community midwives and general practitioners. Pregnant women with a concern of DFM usually contact maternity wards directly. Four to fifteen percent of women present to the hospital in late pregnancy with the primary complaint of reduced or absent fetal movements [8,21,22].

The current study was a part of the ongoing, interdisciplinary collaborative effort related to DFM: Fetal Movement Intervention Assessment (Femina), aiming to survey clinical management and initiate quality improvement efforts in Australia & New Zealand [23], the US [24], the United Kingdom [15] and Norway. The information pamphlet provided to expectant mothers by Norwegian health authorities, instructs women to contact a midwife or a physician "if the baby has become very calm, if they feel less movements - a few or no movements from the fetus" [18,20]. In Norway, significant variation has been shown in maternal recall of information received about fetal movement [10]. Further, women who waited >24 hours with reduced or absent movement before contacting healthcare have been shown to be at increased risk for adverse outcomes [22]. Maternal recall of having received information about fetal movement was associated with more frequent concerns, without improving pregnancy outcomes [10].

Variation in clinical practice, as reflected in patient information, may represent increased risk [25]. Quality assurance efforts aimed at health providers (through clinical guidelines) and pregnant women (through uniform information) were implemented in order to increase identification of high-risk pregnancies for optimal observation and treatment. This paper reports the effects of providing uniform information about fetal activity on maternal awareness, behavior, concerns and pregnancy outcomes when DFM was perceived by the mothers. We hypothesised that providing this information would reduce the number of women who delayed reporting DFM to their healthcare provider, in the total population or by the subgroups defined by maternal age [5,26], body mass index (BMI) [5,27], smoking habits [5,28], and maternal country of origin [29]. We also hypothesised that the intervention was associated with improved pregnancy outcomes, overall and/or by the subgroups. The guidelines for health care providers and effects on clinical management are presented elsewhere [30].

## Methods

### The intervention - information on fetal activity and monitoring

Due to limited high level evidence, the brochure of information was developed using a consensus-based approach; by a systematic literature review, and consultation with leading academics in midwifery and obstetrics across all participating hospitals and a group of pregnant women. The brochure, which included a fetal movement chart (a kick chart), was provided at the ultrasound screening assessment in gestational week 17-19, which 98% of the women attend. The brochure covered information on: expected normal fetal activity [31]; differences in perception according to different fetal movements [31], maternal position [32], the inter- and intraindividual variation between fetuses [9], maternal weight [27], and smoking [33]; interpretation of variation of fetal activity; instructions on how to use the kick chart; and when to contact health professionals if experiencing DFM [11].

Women were informed that their subjective assessment of a decrease in fetal activity was the most important marker of DFM - taking priority over any formal DFM alarm limits [8]. They were instructed not to wait until the next day if they perceived complete absence of fetal activity or if they felt a significant and sustained decrease. If in doubt, as a "thumb rule", they were advised in accordance with the most validated definition for focused counting [11,34]: that a healthy baby very rarely has less than 10 movement in the course of two hours when it usually is active [35]. The brochure was available in Norwegian (Additional file 1), English (Additional file 2), Urdu (Additional file 3), Somali (Additional file 4), Turkish (Additional file 5) and Arabic (Additional file 6). The kick chart was suggested as a supportive tool for women who wished to use it. A modified "count-to-ten" chart [11,36] was chosen, as this has the highest compliance and acceptance rates [4,37,38]. Use of a kick chart is exemplified in additional file 7.

To assist in the clinicians' implementation of this brochure, written information and newsletters were distributed to participating hospitals and regular meetings between clinicians and the study staff were arranged.

### Data collection

Fourteen hospitals across both urban and rural districts, with a total of approximately 33, 000 births annually, were included in the before-and-after study. Two different data collection methods were used pre- and post-intervention: 1) Prospective data collection for women presenting with DFM (DFM population), and 2) Cross-sectional studies (Cross-sectional population):

1) Prospective data collection for all women with singleton pregnancies of ≥28 weeks of gestation presenting at the hospital with a concern of DFM was undertaken by the caregiver without maternal consent and forwarded as anonymous data to the study coordinating centre. Data were collected on maternal demographic characteristics, delay in reporting DFM, clinical management of DFM and pregnancy outcome. Following baseline data collection over a seven month period from April to October 2005, post-intervention data were collected for the 16 month period from November 2005 to March 2007.

2) Cross-sectional studies were performed; pre-intervention (June 2005) and post-intervention (February 2007). Women who birthed at one of the participating hospitals completed a survey anonymously prior to hospital discharge. Further description of this data collection is presented elsewhere [10]. The sample size for the cross-sectional studies was weighted according to number of births in the respective hospitals during the study period.

In addition, population-based data were obtained from the Medical Birth Registry Norway [39] for the purpose of comparisons of the covariates in the study populations versus the total population deliveries in the area. The studies were approved by The Regional Committees for Medical Research Ethics and The Norwegian Data Inspectorate.

### Outcome measures

#### Primary outcome measure

The primary outcome measure was *maternal behavior *in relation to reporting perceived absence or decreased fetal movement to the health provider; defined as the rate of women waiting ≥25 hours with absent fetal movement or ≥48 hours with DFM [6,16,29,40,41].

#### Secondary outcome measures

• *Maternal awareness*: maternal self-report of attention paid to fetal activity.

• *Maternal concerns*: maternal self-report of the frequency of concerns about DFM; dichotomized into being concerned "twice or more" versus "once or never".

• *Receiving information*: maternal self-report of receiving information about fetal activity.

• *DFM consultation*: a consultation at the hospital because of maternal perception of DFM.

• *Pregnancy outcome *for women with DFM was stillbirth; and, for the cross-sectional population; small for gestational (SGA) <10^th ^centile (customized) [42] and emergency cesarean section.

• *Counting group*: proportion of women reporting using a kick chart more than once per week.

Effectiveness in distribution of information and maternal internalization of information were assessed by combining cross-sectional data with the stillbirth rate at hospital levels. As a proxy for effectiveness in distribution, we compared the hospital specific percentage of women reporting receipt of the written information from the cross-sectional surveys with the stillbirth rate in the DFM population. As a proxy for internalization of the information, the percentage for women reporting having used the kick chart twice a week or more was compared with the stillbirth rate in the DFM population.

### Analyses

Statistical analyses were performed in SPSS 14.0.1 (SPSS Inc., Chicago, IL). Crude and adjusted odds ratios (ORs) with 95% confidence intervals (CIs) were estimated, and variables with associations with a p < 0.20 in univariate analyses were included in the multivariate models [43]. Chi square tests were used for estimating differences between proportions of categorical variables. A p-value <0.05 was considered statistically significant. Bonferroni corrections were performed in the multiple comparisons. Subgroup analyses were undertaken according to: maternal age [5,26], body mass index (BMI) [5,27], smoking habits [5,28], and maternal country of origin [29] and according to subgroups of Western and non-Western origin (due to higher rates of stillbirths among non-Western women in our community) [29]. Western mothers were defined as women with origin in Western Europe, North America and Oceania. For women with more than one episode of reporting DFM, only the first episode was included in the analyses.

## Results

**Figure 1:**
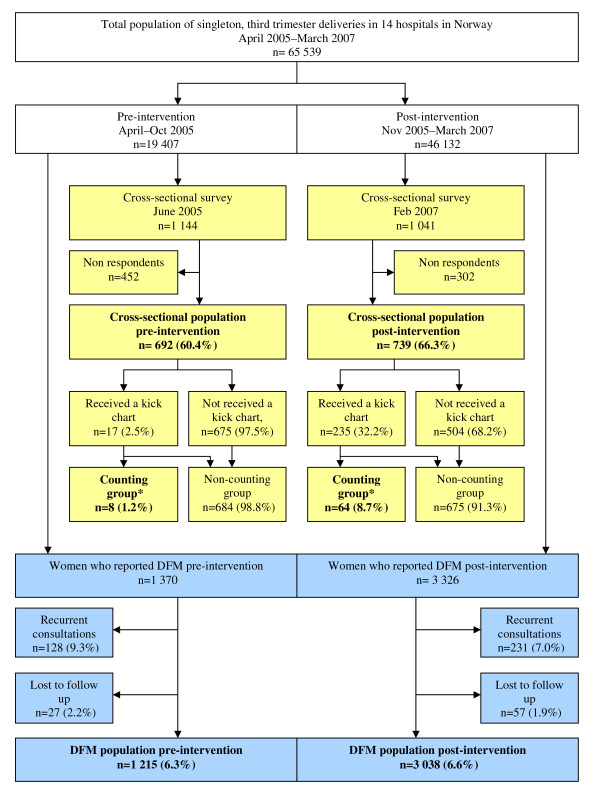
**Trial profile**. Trial profile of total births, reports of decreased fetal movements and the cross-sectional population pre- and post intervention. *Counting group: women reporting using a kick chart more than once per week. Yellow boxes: The cross-sectional surveys Blue boxes: The prospective registrations of DFM consultations

Overview data collection is presented in Figure 1. Baseline characteristics of the populations are described in Table 1. The respondents in the cross-sectional studies were representative for the pregnant population in their area during the study period in regard to age, parity and smoking habits (data from the Medical Birth Registry Norway, not shown).

**Table 1:** Descriptive characteristics: DFM and Cross-sectional populations

	DFM* N = 4 253			Cross-sectional* N = 1 431		
**Characteristics**	**Pre-intervention** **n = 1 215** **n (%)†**	**Post-intervention** **n = 3 038** **n (%)†**	***P*‡**	**Pre-intervention** **n = 692** **n (%)†**	**Post-intervention** **n = 739** **n (%)†**	***P*‡**

**Age, y mean (SD)**	29.6 (4.9)	29.7 (5.2)	0.625	30.2 (4.9)	30.1 (5.1)	0.849
<20	23 (1.9)	59 (2.0)		9 (1.3)	10 (1.4)	
20-24	182 (15.1)	454 (15.1)		70 (10.3)	101 (13.7)	
25-29	388 (32.3)	933 (31.1)		231 (34.0)	208 (28.1)	
30-34	413 (34.4)	1 031 (34.3)		237 (34.9)	273 (36.9)	
35+	196 (16.3)	527 (17.5)		133 (19.6)	147 (19.9)	

**Parity**						
Para 0	559 (51.1)	1 414 (52.4)	0.490	287 (43.1)	300 (41.4)	0.197
Para 1	372 (34.0)	878 (32.5)		221 (33.2)	283 (39.0)	
Para 2+	163 (14.9)	409 (15.2)		158 (23.7)	142 (19.5)	

**BMI, kg/m^2^**	24.7 (5.1)	24.5 (5.0)	0.547	24.4 (4.4)	23.6 (4.2)	<0.001
<20	143 (13.3)	383 (14.2)		74 (11.0)	113 (15.6)	
20-24	547 (50.8)	1325 (49.0)		378 (56.2)	412 (56.9)	
25-29	244 (22.7)	638 (23.6)		147 (21.8)	137 (18.9)	
30+	91 (8.5)	249 (9.2)		74 (11.0)	21 (8.6)	

**Smoking habits**						
Smoking	104 (8.8)	259 (8.9)	0.924	50 (7.4)	48 (6.4)	0.483

**Country of origin**						
Non-Western	178 (14.7)	406 (13.4)	0.271	39 (5.7)	29 (3.6)	0.064

* Data are reported as n(%) unless otherwise noted.

† Denominators vary due to missing values

‡ Chi square tests for the difference between proportions within women with DFM and the cross-sectional population respectively

### Information and maternal awareness of fetal activity

Data from the cross-sectional studies showed that one in four women did not recall receiving information about normal expected fetal activity by their health provider, both pre- and post-intervention. Recall of receiving information was associated with higher awareness of fetal activity, both pre-intervention (OR 2.0, 95% CI 1.2-3.3) and post-intervention (OR 1.8, 95% CI 1.0-3.1, p = 0.043). Pre-intervention, recall of receiving information was associated with more frequent maternal concern (OR 1.7, 95% CI 1.2-2.4); while this association was not longer present post-intervention (OR 1.3, 95% CI 0.9-1.9).

Maternal recall of information about limits for normality was more homogeneous in the intervention period, e.g. 22% recalled having seen the thumb rule (10 kicks in two hours) at baseline measurement, versus 42% in the intervention period (p = 0.022). Pre-intervention, low maternal awareness to fetal activity was associated with an increased risk of having an SGA baby; [10] this association was not observed in the post-intervention period (OR 1.3, 95% CI 0.6-2.9).

### Maternal behavior and pregnancy outcomes

Among women with DFM, the stillbirth rate was lower in post-intervention period; 4.2% versus 2.4% (Tveit et al, submitted 2009). The reduction in stillbirth was isolated to primiparous women only. Primiparous women also reported DFM earlier than all other women included (Table 2). In the total population, the mean gestational age at the time of reporting DFM was two days lower during the post-intervention period; 36^6 ^versus 36^4 ^weeks, p = 0.006.

**Table 2:** DFM population: Effects of intervention on maternal behavior and stillbirth rates, stratified by subgroups

	Overall N = 4 253		Stratified by subgroups n (%)									
	**Pre-intervention** **n = 1 215** **n (%)**	**Post-intervention** **n = 3 038** **n (%)**	**Primiparous** **n = 1 973** **(52.0)**	**P-value**	**≥ 35 years** **n = 724** **(17.2)**	**P-value**	**Overweight** **n = 1 400** **(36.8)**	**P-value**	**Smokers** **n = 363** **(8.9)**	**P-value**	**Non-Western** **n = 583** **(13.7)**	**P-value**

**Consultation**			**Adj OR (95% CI)^§^**									

By own initiative	363 (32.1)	656 (30.8)	1.22 (0.95-1.57)	0.117	1.43 (0.99-2.08)	0.652	1.10 (0.81-1.50)	0.524	0.87 (0.47-1.62)	0.657	0.88 (0.54-1.46)	0.631
At the delivery unit	661 (54.9)	1, 716 (57.4)	**1.35** (1.09-1.67)	0.007	1.01 (0.76-1.34)	0.057	1.24 (0.96-1.60)	0.106	0.82 (0.47-1.44)	0.493	1.06 (0.68-1.67)	0.786
During night (6 pm-8 am)	317 (27.7)	846 (29.9)	**1.39** (1.09-1.77)	0.007	1.31 (0.83-2.07)	0.258	1.41 (1.05-1.89)	0.023	1.78 (0.99-3.21)	0.055	1.08 (0.65-1.80)	0.775
In weekends	258 (21.2)	607 (20.0)	1.04 (0.80-1.35)	0.758	0.84 (0.65-1.69)	0.855	1.28 (0.94-1.76)	0.121	0.51 (0.27-0.96)	0.037	0.88 (0.50-1.52)	0.634
DFM ≥ 48 hrs	415 (53.6)	897 (48.9)	**0.61** (0.47-0.81)	<0.001	0.82 (0.51-1.32)	0.414	1.12 (0.81-1.54)	0.507	1.47 (0.74-2.91)	0.274	0.54 (0.29-0.99)	0.045
Absent FM ≥ 25 hrs	99 (23.9)	201 (18.0)	0.72 (0.47-1.09)	0.117	1.00 (0.43-2.32)	0.996	0.90 (0.55-1.47)	0.668	0.60 (0.22-1.61)	0.309	0.64 (0.29-1.43)	0.274
Fetal deaths	50(4.2)	73 (2.4)	**0.36** (0.19-0.69)	0.002	0.92 (0.35-2.44)	0.902	0.60 (0.30-1.20)	0.151	1.48 (0.40-5.53)	0.559	0.99 (0.25-4.02)	0.993

^† ^Univariate logistic regression analyses with 95% CI, at baseline is the reference category

^§ ^Multivariate logistic regression analyses with 95% CI, adjusting for the covariates (parity, maternal age, BMI, smoking habits, maternal origin)

Denominators vary due to missing values. Bold numbers indicate significant values after Bonferroni correction for multiple comparisons

In the post-intervention group, overweight women in the cross-sectional populations described higher awareness of fetal activity (Table 3). No behavior changes were observed among overweight women if they perceived DFM (Table 2).

**Table 3:** Cross-sectional population: Low maternal awareness of fetal activity and maternal characteristics (N = 1431)*

	Pre-intervention, n = 692			Post-intervention, n = 739		
		**Low maternal awareness†** **n = 78 (11.7%)**			**Low maternal awareness†** **n = 62 (8.9%)**	
	
**Maternal characteristics**	**Values** **n (%)**	**Crude OR** **(95% CI)**	**Adj OR** **(95% CI)**	**Values** **n (%)**	**Crude OR (95% CI)**	**Adj OR** **(95% CI)**

Primiparous (reference: multiparous)	287 (43.1)	**0.57**(0.34-0.96) p = 0.032	0.87 (0.58-1.30) p = 0.494	300 (41.4)	0.98 (0.57-1.67) p = 0.930	Not included
Age ≥ 35 yrs (reference: <35 years old)	133 (19.6)	**2.67**(1.61-4.45) p < 0.001	**1.64**(1.06-2.54) p = 0.026	147 (19.9)	1.38 (0.76-2.51) p = 0.290	1.34 (0.74-2.52) p = 0.316
BMI >25 kg/m^2^ (reference: BMI ≤ 25 kg/m^2^)	221 (32.8)	1.38 (0.84-2.27) p = 0.208	0.77 (0.50-1.18) p = 0.226	158 (27.5)	0.53 (0.27-1.04) p = 0.063	**0.43**(0.21-0.89) p = 0.024
Smokers (reference: non-smokers)	50 (7.4)	0.67 (0.24-2.00) p = 0.503	Not included	48 (6.4)	0.74 (0.22-2.46) p = 0.622	Not included
Non-Western origin (reference: Western origin)	39 (5.7)	**2.54**(1.11-5.83) p = 0.023	1.79 (0.83-3.83) p = 0.226	29 (3.6)	**3.50**(1.34-9.08) p = 0.006	**3.34**(1.27-8.78) p = 0.015

* Detailed results from the baseline population are presented elsewhere [10].

† Univariate and multivariate logistic regression analyses with 95% CI for the associations between the analyzed groups. Denominators vary due to missing values. Bold numbers indicate significant values.

^§^Multivariate logistic regression analyses with 95% CI, adjusting for the covariates (parity, maternal age, BMI, smoking habits, maternal origin)

Pre-intervention, smoking mothers in the cross-sectional population recalled less receipt of information about fetal activity than non-smokers, OR 0.5 (95% CI 0.3-0.9). This association was not present in post-intervention, OR 0.6 (95% CI 0.3-1.2). No changes in maternal behavior were observed among smoking women perceiving DFM (Table 2).

Non-Western women in the cross-sectional study post-intervention, remained the only risk group reporting both less receipt of information (adjusted OR 0.4, 95% CI 0.2-0.8) and low awareness of fetal activity (Table 3). Among the non-Western women who perceived DFM, the intervention showed no changes in maternal behavior, frequency of concerns or outcomes (Table 2).

The hospital-specific percentage of women reporting having received written information (proxy for distribution) was negatively associated with mortality rates - the more information, the lower mortality (β = 0.974, p = 0.031). This was done to assess the effect of the distribution of information and maternal internalization of it on the number of stillbirths.

### Maternal concerns - as reported by women in the cross-sectional studies

Mothers in the post-intervention period did not report concerns or have a DFM consultation more frequently (Table 4). Overweight women were the only subgroup reporting increased concerns; however, this was not significant after Bonferroni correction (Table 4). When concerned, the mothers more often related their concern to the fetal activity level earlier in the actual pregnancy (44% vs. 51%, p = 0.011). More often, the concerned mothers assessed their perception of DFM *not *being normal for their baby and that their concern was a true reason for being concerned (28% vs. 33%, p = 0.022). Being concerned was associated with being examined at hospital both pre-intervention (OR 4.9, 95% CI 3.0-7.8) and post-intervention (OR 5.8, 95% CI 3.7-9.2).

**Table 4:** Cross-sectional population: Effects of intervention on maternal awareness, concern and maternal behavior (N = 1431)

	Overall N = 1 431				Stratified by subgroups post-intervention, n = 739 n (%)*				
	**Pre-intervention** **n = 692** n (%)*	**Post-intervention** **n = 715** n (%)*	**Crude OR** **(95% CI)^†^**	**Adjusted OR** **(95% CI)^§^**	**Primiparous** **n = 587 (57.8)**	**≥ 35 years** **n = 280 (19.7)**	**Overweight** **n = 421 (30.1)**	**Smokers** **n = 97 (6.9)**	**Non-Western** **n = 67 (4.7)**

					**Adj OR (95% CI)^§^**				

Low awareness	78 (11.7)	62 (8.9)	0.72 (0.50-1.02) p = 0.060	0.84 (0.57-1.24) p = 0.356	1.19 (0.63-2.26) p = 0.585	0.49 (0.24-1.00) p = 0.050	0.44 (0.20-1.00) p = 0.051	0.74 (0.15-3.68) p = 0.712	1.10 (0.23-5.27) p = 0.909
Concerned	341 (50.7)	417 (57.9)	1.15 (0.93-1.42) p = 0.210	1.20 (0.96-1.50) p = 0.114	0.95 (0.68-1.34) p = 0.783	1.32 (0.80-2.19) p = 0.273	1.54 (1.03-2.31) p = 0.037	0.98 (0.42-2.34) p = 0.969	3.21 (1.04-9.93) p = 0.043
DFM consultation	98 (14.2)	122 (16.4)	1.18 (0.88-1.57) p = 0.263	1.32 (0.97-1.78) p = 0.075	1.38 (0.88-2.17) p = 0.163	1.10 (0.54-2.21) p = 0.801	1.06 (0.93-2.63) p = 0.092	1.32 (0.46-3.80) p = 0.613	0.42 (0.10-1.82) p = 0.245

* Denominators vary due to missing values.

† Univariate regression analyses with 95% CI for the associations between the analyzed groups.

^§ ^Multivariate logistic regression analyses with 95% CI, adjusting for the covariates (parity, maternal age, BMI, smoking habits, maternal origin).

### Fetal movement counting in the intervention group

In the post-intervention group, 235 (32%) reported using a kick chart, as opposed to 8 (1%) pre-intervention. Post-intervention, 64 (9%) of women used a kick chart more than once per week (*counting group*); versus 8 (1%) pre-intervention. Primiparous women were more likely than multiparous women to use a kick chart more than once per week (OR 2.3, 95% CI 1.3-4.2). No non-Western mothers used a kick chart.

Maternal experiences with use of a kick chart in the intervention period are presented in Table 5, illustrating the benefits of maternal receipt of receiving information on *how *and *why *to use the kick chart. The use of a kick chart was not associated with increased maternal concerns about DFM (32% in the *non-counting group *vs. 42% in the *counting group*, p = 0.090). Use of a kick chart was associated with a reduced risk of having a DFM consultation, 18% vs. 9% (p = 0.045). One of ten babies was SGA in both groups. Eleven (7%) of the *non-counting group *had an emergency caesarean section, as opposed to one (2%) in the *counting group *(p = 0.047).

**Table 5:** Cross-sectional population post-intervention: Experiences with use of a kick chart (N = 235)*

Maternal experiences with use of a kick cart	n (%)	Recalled receipt of information about HOW to use the kick chart n = 119 (63.3%) OR (95% CI)†	P value	Recalled receipt of information about WHY use the kick chart n = 121 (66.5%) OR (95% CI) †	P value
Kick counting was time-consuming	97 (41.3)	0.3 (0.1-0.7)	0.003	0.4 (0.2-0.9)	0.039
Kick counting stimulated to "get to know" the baby	71 (30.2)	2.2 (0.9-5.4)	0.099	1.7 (0.7-4.3)	0.251
Appreciated the visual presentation of the fetal activity	74 (31.5)	3.3 (1.3-8.4)	0.011	1.9 (0.7-4.9)	0.189
Kick counting induced too much focus on fetal activity	47 (20.0)	1.2 (0.5-3.0)	0.568	0.8 (0.4-1.9)	0.727

* Denominators vary due to missing values.

† Univariate regression analyses with 95% CI for the associations between the analyzed groups. Reference groups: women who did not recall receipt of information about *how *to and *why *use of a kick chart respectively.

The hospital-specific percentage of women reporting having used the kick chart more than once per week or more (proxy for internalization) was negatively associated with mortality (β = 0.922, p = 0.005). This does not reflect the effect of kick counting on an individual level, as there are no data to support this, only the benefit of effective information.

## Discussion

In this prospective before-and-after study, primiparous women were shown to have the greatest behavioral change in reporting DFM and were the only risk group with a reduction in stillbirth. This may be associated with the experience of transition to the motherhood role of first-time mothers. With no previous experiences, pregnancy represents a major adjustment period, strongly influenced by information seeking and trying to adopt best health practices and changes in lifestyle [44].

While the effect of printed educational materials as guidelines for health care providers is associated with some improvement in process of care [45], the addition of additional interventions such as outreach education and audit and feedback may enhance this effect [46]. In this study, implementation of standardized information for women across participating hospitals was achieved through a multifaceted intervention including clinical practice recommendations, outreach education and audit and feedback. Standardized written information improved maternal self-screening of significance for decrease in or absence of fetal movement. This may have contributed to the decreased stillbirth rate among primiparous women. The importance of recognizing DFM for pregnancy outcomes is indisputable [2,4,22], and identification of risk is one of the main goals for antenatal care [47]. Women were advised to contact their health care provider for concerns about DFM regardless of reaching in any specific fetal movement rate threshold. The advice on focused counting and the suggested "alarm limits" [7,11] when women were in doubt about the presence of DFM in addition to the advice about their perceptions may have contributed to a reduction in excessive delay in reporting DFM.

A similar proportion (75%) of women recalled having received information in the baseline and the intervention. Thus, this provides support to the effectiveness of the information to improve maternal self-screening of DFM which was more explicit than the previous information [18] and emphasized maternal assessment of fetal activity according to the activity pattern for her *own *child [9]. This was reflected in the mothers' reasoning for concern in the post-intervention. Women in the post-implementation period reported concerns related to the activity level earlier in the pregnancy more often and were more confident that their perception of DFM was the true reason for being concerned.

### Overweight mothers - higher awareness, more concerns, but not improved pregnancy outcomes

Being overweight increases the risk of not perceiving DFM (Tveit et al, submitted 2009). However, it is unknown whether reduced perception of fetal movements among overweight women is due to higher risk of a true decrease in fetal movement or to a lower ability to perceive fetal activity [27]. In the post-intervention period, overweight women described higher awareness of fetal activity, they more frequently reported concerns of DFM and presented at the hospital during the night more frequently. However, no difference was shown in the excessive delay in reporting of decreased or absent fetal movement or in the stillbirth rate among these mothers.

### Mothers of non-Western origin - less access to information

In the non-Western population the intervention was not associated with changes in maternal behavior or the stillbirth rates. Non-Western women had three times increased risk of low awareness of fetal activity when compared to the Western mothers, and were shown to have the lowest rates of receiving the information about expected fetal activity, in spite of available information brochures in the most common foreign languages in the area. This may be due to the presentation of the information not adequately meeting their needs or to cultural differences in risk orientation [48]. Communication problems between non-Western women and health care providers have been identified as a risk factor for adverse pregnancy outcomes [29,48]. This confirms the need for a greater focus on providing culturally appropriate information which is written at an appropriate level to ensure comprehensive uptake particularly for those women at increased risk [49].

The population non-Western women in our study were mainly from low-income countries and a wide variation in cultures was represented. Minority or marginalized women in a high-income country do not appear to display a "healthism" approach to their lives [50]; normative assumptions in antenatal guidelines do not apply. This may be in part due to a lack of trust in caregivers among minority women in Western countries [50,51], the authoritative source often are their husband [51] or their mother [52], instead of the health care services. While printed educational materials are widely used to improve knowledge, awareness and attitudes, especially in developed countries, other methods for information and education may be needed for cultural minority groups. The impact of life style choices and compliance to recommendations from health providers may be higher if role models and authoritative sources, such as the husband and/or mother, are involved in the antenatal care. Further research is needed on appropriate methods to change health seeking behavior in pregnancy, including DFM, for non-Western women in our setting.

### Fetal movement counting associated with well-being and safety

While the majority of women chose not to use a kick chart, its use was associated with less maternal concerns, as well as a reduced risk of being examined in hospital because of DFM. Satisfaction with the information about the rationale for fetal monitoring and the technique of recording were associated with more frequent use of a kick chart and increased the mothers' assessment that a kick chart was important and useful. Effective communication specific for each woman's need and encouragement by a consistent healthcare professional have been identified as the key factor for high compliance for use of a kick chart [6,31,53].

Many health professionals do not to recommend a FMC in their low-risk patients because they fear it will cause increased maternal concern and anxiety [54], as well as increased unnecessary consultations and/or interventions [7,55]. The current study was not a study to evaluate the use of kick chart per se. Nevertheless, it is important to notice that use of a kick chart was not associated with increased concerns or more frequent consultations in the hospital. It seemed to be "safe" with regard to maternal well-being and use of health resources. We have no evidence that FMC with specific alarm limits are preferable or superior to subjective maternal opinion. However, previous reports also indicate that the use of a kick chart does not cause anxiety or other adverse psychological effects [54,56,57]. Further research is needed, both in low- and high-risk populations [4,12].

### Methodological considerations

The true effect of such an intervention may be better estimated using a randomized trial methodology. However, in a quality improvement setting like ours, a before-and-after study design was chosen. There are potential problems in using RCT to test the effect of information within the same population due to the likelihood of contamination. While the before-and-after study design may overestimate the true effect, the prospective nature of this study may limit this effect. Additional methodological considerations are presented in the article from the other part of the quality improvement, the clinical management of DFM pregnancies [30]. Potential recall bias and the validity of the cross-sectional questionnaire have been discussed elsewhere [10].

While these findings are encouraging, caution in its interpretation is warranted due to limitations of the design employed in this quality improvement project; the implemented solutions were based on the local existing imperfections found by prior data collections of quality indicators. The results may thus not be directly transferable to other populations. Yet, reports from a variety of locations suggest that significant variability in the information given to expecting women is a wide-spread quality issue in obstetric care [15,22,23,58].

## Conclusions

Uniform information about fetal activity provided to pregnant women was associated with a reduction in the number of primiparous women who delayed reporting of DFM and reduced stillbirth rates for primiparous women reporting DFM. The information did not appear to increase maternal concerns or frequency of consultations. While these findings are encouraging, caution in its interpretation is warranted due to limitations of the design employed in this quality improvement project; the implemented solutions were based on the local issues identified by prior quality assurance studies. Further studies replicating these findings are required. A clearer definition of DFM is needed.

## Competing interests

The authors declare that they have no competing interests.

## Authors' contributions

ES: Design of the study, data collection, analysis, interpretation of data, writing and finalizing the manuscript. JVHT: Design of the study, data collection, interpretation of data and revising the manuscript. VF: Design of the study, interpretation of data, writing and revising the manuscript. BSP: Design of the study, interpretation of data and revising the manuscript. PEB: Design of the study. RF: Design of the study, interpretation of data and revising the manuscript. JFF: Design of the study, analysis, interpretation of data, writing and revising the manuscript.

All authors have approved the final version of the manuscript.

## Supplementary Material

Additional file 1 **Kick Count.** Kicks Count brochure, Norwegian version. A brochure of information aiming to increase maternal awareness and vigilance to significant decreases in fetal activity, and to aid health promoting behavior. The brochure was provided as a part of the routine information given to women at the standard ultrasound assessment at 17-19 weeks in Norway as a part of the quality improvement intervention. Click here for file

Additional file 2 **Kick Count. **Kicks Count brochure, English version. A brochure of information aiming to increase maternal awareness and vigilance to significant decreases in fetal activity, and to aid health promoting behavior. The brochure was provided as a part of the routine information given to women at the standard ultrasound assessment at 17-19 weeks in Norway as a part of the quality improvement intervention. Click here for file

Additional file 3 **Kick Count. **Kicks Count brochure, Urdu version. A brochure of information aiming to increase maternal awareness and vigilance to significant decreases in fetal activity, and to aid health promoting behavior. The brochure was provided as a part of the routine information given to women at the standard ultrasound assessment at 17-19 weeks in Norway as a part of the quality improvement intervention. Click here for file

Additional file 4 **Kick Count. **Kicks Count brochure, Somali version. A brochure of information aiming to increase maternal awareness and vigilance to significant decreases in fetal activity, and to aid health promoting behavior. The brochure was provided as a part of the routine information given to women at the standard ultrasound assessment at 17-19 weeks in Norway as a part of the quality improvement intervention. Click here for file

Additional file 5 **Kick Count. **Kicks Count brochure, Turkish version. A brochure of information aiming to increase maternal awareness and vigilance to significant decreases in fetal activity, and to aid health promoting behavior. The brochure was provided as a part of the routine information given to women at the standard ultrasound assessment at 17-19 weeks in Norway as a part of the quality improvement intervention. Click here for file

Additional file 6 **Kick Count. **Kicks Count brochure, Arabic version. A brochure of information aiming to increase maternal awareness and vigilance to significant decreases in fetal activity, and to aid health promoting behavior. The brochure was provided as a part of the routine information given to women at the standard ultrasound assessment at 17-19 weeks in Norway as a part of the quality improvement intervention. Click here for file

Additional file 7 **Example of use of a kick chart**. An example of a kick chart used by a women participating in the study Click here for file

## References

[B1] Sinha D Obstetric outcome in women complaining of reduced fetal movements J Obstet Gynaecol 2007 27 41 43 10.1080/01443610601016909 17365457

[B2] Olesen AG Svare JA Decreased fetal movements: background, assessment, and clinical management Acta Obstet Gynecol Scand 2004 83 818 26 15315592 10.1111/j.0001-6349.2004.00603.x

[B3] Heazell AE Sumathi GM Bhatti NR What investigation is appropriate following maternal perception of reduced fetal movements? J Obstet Gynaecol 2005 25 648 650 10.1080/01443610500278303 16263536

[B4] Mangesi L Hofmeyr GJ Fetal movement counting for assessment of fetal wellbeing (Review) 2007 1 John Wiley & Sons, Ltd 10.1002/14651858.CD004909.pub2 17253530

[B5] Frøen JF others Risk factors for sudden intrauterine unexplained death: Epidemiologic characteristics of singleton cases in Oslo, Norway, 1986-1995 Am J Obstet Gynecol 2001 184 694 702 10.1067/mob.2001.110697 11262474

[B6] Grant A others Routine formal fetal movement counting and risk of antepartum late death in normally formed singletons Lancet 1989 2 345 349 10.1016/S0140-6736(89)90535-7 2569550

[B7] Heazell AEP Frøen JF Methods of fetal movement counting and the detection of fetal compromise J Obstet Gynaecol 2008 28 147 154 10.1080/01443610801912618 18393008

[B8] Frøen JF Fetal movement assessment Semin Perinatol 2008 32 243 246 10.1053/j.semperi.2008.04.004 18652921

[B9] Groome LD Swiber MJ Holland SB Bentz LS Atterbury JL Trimm RF 3rd Spontaneous motor activity in the perinatal infant before and after birth, stability in individual differences Dev Psychobiol 1999 35 1 25 34 10.1002/(SICI)1098-2302(199907)35:1<25::AID-DEV4>3.0.CO;2-Q 10397892

[B10] Saastad E Ahlborg T Frøen JF Low maternal awareness of fetal movement is associated with small for gestational age infants J Midwifery Womens Health 2008 53 345 352 10.1016/j.jmwh.2008.03.001 18586188

[B11] Moore TR Piacquadio K A Prospective Evaluation of Fetal Movement Screening to Reduce the Incidence of Antepartum Fetal Death Am J Obstet Gynecol 1989 160 1075 1080 2729383 10.1016/0002-9378(89)90164-6

[B12] Haws RA Reducing stillbirths: screening and monitoring during pregnancy and labour BMC Pregnancy Childbirth. 2009 9 Suppl 1 S5 10.1186/1471-2393-9-S1-S5 PMC2679411 19426468

[B13] Neldam S Fetal movements as an indicator of fetal well-being Dan Med Bull 1983 30 274 8 6872585

[B14] Berbey R Manduley P De-Vigil G Counting fetal movements as a universal test for fetal wellbeing International Journal of Gynaecology & Obstetrics 2001 74 293 295 10.1016/S0020-7292(01)00438-6 11543756

[B15] Heazell AE Midwives' and obstetricians' knowledge and management of women presenting with decreased fetal movements Acta Obstet Gynecol Scand. 2008 87 3 331 339 10.1080/00016340801902034 18307074

[B16] Frøen JF A kick from within-fetal movement counting and the cancelled progress in antenatal care J Perinat Med 2004 32 13 24 10.1515/JPM.2004.003 15008381

[B17] Guidelines for perinatal care 2002 5 Elk Grove Village, IL & Washington DC: American Academy of Pediatrics, The American College of Obstetricians and Gynecologists

[B18] Sosial- og helsedirektoratet Retningslinjer for svangerskapsomsorgen [Guidelines for antenatal care]. [Norwegian] 2005 Oslo: Sosial- og Helsedirektoratet

[B19] RCOG Antenatal care - routine care for the healthy pregnant woman 2003 London: RCOG Press 21370514

[B20] Kohner N others The Pregnancy Book 2007 7 London: The Department of Health, UK

[B21] Sergent F [Decreased fetal movements in the third trimester: what to do?] Gynecol Obstet Fertil 2005 33 861 869 10.1016/j.gyobfe.2005.07.041 16243568

[B22] Frøen JF [Clinical practice variation in reduced fetal movements] Tidsskr Nor Laegeforen 2005 125 2631 2634 16215607

[B23] Flenady V MacPhail J Gardener G Chadha Y Mahomed K Egan S Heazell AE Fretts RC Frøen JF Management of decreased fetal movements in Australia and New Zealand: A survey of practice Perinatal Society of Australia and New Zealand 9th Annual Congress 2006

[B24] Fretts R Frøen JF Cavanagh E Reynolds D A prospective study of pregnancies with Decreased Fetal Movement3rd Annual Conference of the International Stillbirth Alliance 2007

[B25] Safe Practices for Better Healthcare: A Consensus Report. 2003 2009 Washington, DC: The National Quality Forum

[B26] Fretts RC Etiology and prevention of stillbirth Am J Obstet Gynecol 2005 193 1923 1935 10.1016/j.ajog.2005.03.074 16325593

[B27] Sebire NJ Maternal obesity and pregnancy outcome: a study of 287 213 pregnancies in London International Journal of Obesity & Related Metabolic Disorders 2001 25 1175 82 10.1038/sj.ijo.0801670 11477502

[B28] Coppens M Computerized analysis of acute and chronic changes in fetal heart rate variation and fetal activity in association with maternal smoking Am J Obstet Gynecol 2001 185 421 426 10.1067/mob.2001.115992 11518903

[B29] Saastad E Vangen S Frøen JF Suboptimal care in stillbirths - a retrospective audit study Acta Obstet Gynecol Scand 2007 86 444 450 10.1080/00016340701207724 17486466

[B30] Tveit JV Reduction of late stillbirth with the introduction of fetal movement information and guidelines - a clinical quality improvement BMC Pregnancy and Childbirth 2009 9 32 10.1186/1471-2393-9-32 19624847 PMC2734741

[B31] Velazquez MD Rayburn WF Antenatal Evaluation of the Fetus Using Fetal Movement Monitoring Clin Obstet Gynecol 2002 45 993 1004 10.1097/00003081-200212000-00006 12438877

[B32] Cito G Maternal position during non-stress test and fetal heart rate patterns Acta Obstet Gynecol Scand 2005 84 335 338 15762962 10.1111/j.0001-6349.2005.00644.x

[B33] Graca LM Acute effects of maternal cigarette smoking on fetal heart rate and fetal body movements felt by the mother J Perinat Med 1991 19 385 390 10.1515/jpme.1991.19.5.385 1804949

[B34] American Academy of Pediatrics, The American College of Obstetricians and Gynecologists Guidelines for perinatal care 2002 5 Washington, DC: AAP and ACOG

[B35] Frøen JF Management of Decreased Fetal Movements Semin Perinatol 2008 32 307 311 10.1053/j.semperi.2008.04.015 18652933

[B36] Pearson JF Fetal movement recording: a guide to fetal well-being Nurs Times 1979 75 1639 1641 258354

[B37] Christensen FC Olson K Rayburn WF Cross-over trial comparing maternal acceptance of two fetal movement charts Journal of Maternal-Fetal & Neonatal Medicine 2003 14 118 122 10.1080/jmf.14.2.118.122 14629093

[B38] Gomez LM Compliance with a fetal movement chart by high-risk obstetric patients in a Peruvian hospital Am J Perinatol 2007 24 89 93 10.1055/s-2006-958160 17268946

[B39] The Medical Birth Registry of Norway 2007 http://www.fhi.no/eway/default.aspx?pid=233&trg=MainArea_5661&MainArea_5661=5665:0:15.3278:1:0:0:::0

[B40] Sadovsky E The definition and the significance of decreased fetal movements Acta Obstet Gynecol Scand 1983 62 409 413 10.3109/00016348309154211 6666553

[B41] Harrington K Obstetric outcome in women who present with a reduction in fetal movements in the third trimester of pregnancy J Perinat Med 1998 26 77 82 10.1515/jpme.1998.26.2.77 9650126

[B42] Gardosi J Customized fetal growth standards: rationale and clinical application Seminars in Perinatology 2004 28 33 40 10.1053/j.semperi.2003.12.002 15058900

[B43] Hosmer D Lemeshow S Applied Logistic Regression 2000 2 John Wiley & Sons Inc full_text

[B44] Deave T Johnson D Ingram J Transition to parenthood: the needs of parents in pregnancy and early parenthood BMC Pregnancy and Childbirth 2008 8 30 10.1186/1471-2393-8-30 18664251 PMC2519055

[B45] Farmer AP Printed educational materials: effects on professional practice and health care outcomes Cochrane Database of Systematic Reviews 2008 CD004398 10.1002/14651858.CD004398.pub2 18646106

[B46] Grimshaw JM Effectiveness and efficiency of guideline dissemination and implementation strategies Health Technology Assessment (Winchester, England) 2001 8 iii iiv 10.3310/hta8060 14960256

[B47] Chalmers B Mangiaterra V Porter R WHO principles of perinatal care: the essential antenatal, perinatal, and postpartum care course Birth 2001 28 202 207 10.1046/j.1523-536x.2001.00202.x 11552969

[B48] Essen B others Are some perinatal deaths in immigrant groups linked to suboptimal perinatal care services? BJOG: an International Journal of Obstetrics & Gynaecology 2002 109 677 682 12118647

[B49] Freda MC Issues in patient education Journal of Midwifery & Women's Health 2004 49 203 209 15134673 10.1016/j.jmwh.2004.01.003

[B50] Downe S 'Weighing up and balancing out': a meta-synthesis of barriers to antenatal care for marginalised women in high-income countries BJOG: An International Journal of Obstetrics and Gynaecology 2009 116 518 529 10.1111/j.1471-0528.2008.02067.x 19250363

[B51] Ny P Middle Eastern mothers in Sweden, their experiences of the maternal health service and their partner's involvement Reproductive Health 2007 4 9 10.1186/1742-4755-4-9 17958884 PMC2173883

[B52] Burton LM Age norms, the timing of family role transitions, and intergenerational caregiving among aging African American women The Gerontologist 1996 36 199 8920089 10.1093/geront/36.2.199

[B53] Kuwata T Establishing a reference value for the frequency of fetal movements using modified "count to 10" method J Obstet Gynaecol Res 2008 34 318 323 10.1111/j.1447-0756.2008.00791.x 18588609

[B54] Hill-Smith I Professional and patient perspectives of NICE guidelines to abandon maternal monitoring of fetal movements Br J Gen Pract 2004 54 858 861 15527614 PMC1324922

[B55] Flenady V Gardener G MacPhail J Chadha Y King J Cole S McCowan LF Frøen JF Fetal Movement Monitoring: Practice in Australia and New Zealand The Perinatal Society of Australia and New Zealand, 9th Annual Congress, Perth 2006

[B56] Liston RM Bloom K Zimmer P The psychological effects of counting fetal movements Birth 1994 21 135 140 10.1111/j.1523-536X.1994.tb00512.x 7857455

[B57] Mikhail MS The effect of fetal movement counting on maternal attachment to fetus American Journal of Obstetrics & Gynecology 1991 165 988 991 10.1016/0002-9378(91)90455-z 1951568

[B58] Saastad E Frøen JF [Reduced fetal movements--clinical management, recommendations and information] Tidsskr Nor Laegeforen 2005 125 2627 2630 16215606

