# Supplementary material for: Implementation of uniform information on fetal movement in a Norwegian population reduced delayed reporting of decreased fetal movement and stillbirths in primiparous women - a clinical quality improvement
Source: BMC Res Notes. 2010 Jan 4;3:2. doi: 10.1186/1756-0500-3-2 (PMC2837670; doi:10.1186/1756-0500-3-2)
Supplement: Additional file 7 — Example of use of a kick chart. An example of a kick chart used by a women participating in the study [file 1756-0500-3-2-S7.PDF]

Note: Tick the box with a pen

I start counting today (date, dd.mm.yy) 12.04.07 My due date is (dd.mm.yy) 06.07.07 I normally count between (time): 18.30 and 20.30

WEEKDAYS

MINUTES (START COUNTING; 5 minutes per box)

WEEK 28

Minutes	Day 1	Day 2	Day 3	Day 4	Day 5	Day 6	Day 7
0-5		X					
6-10	X			X		X	X
11-15			X		X		
16-20							
21-25							
26-30							
31-35							
36-40							
41-45							
46-50							
51-55							
56-60							
61-65							
66-70							
71-75							
76-80							
81-85							
86-90							
91-95							
96-100							
101-105							
106-110							
111-115							
116-120							
Over 2 hours							

WEEK 29

Minutes	Day 1	Day 2	Day 3	Day 4	Day 5	Day 6	Day 7
0-5	X	X					X
6-10			X		X	X	
11-15				X			
16-20							
21-25							
26-30							
31-35							
36-40							
41-45							
46-50							
51-55							
56-60							
61-65							
66-70							
71-75							
76-80							
81-85							
86-90							
91-95							
96-100							
101-105							
106-110							
111-115							
116-120							
Over 2 hours							

WEEK 30

Minutes	Day 1	Day 2	Day 3	Day 4	Day 5	Day 6	Day 7
0-5				X		X	X
6-10		X			X		
11-15	X		X				
16-20							
21-25							
26-30							
31-35							
36-40							
41-45							
46-50							
51-55							
56-60							
61-65							
66-70							
71-75							
76-80							
81-85							
86-90							
91-95							
96-100							
101-105							
106-110							
111-115							
116-120							
Over 2 hours							

WEEK 31

Minutes	Day 1	Day 2	Day 3	Day 4	Day 5	Day 6	Day 7
0-5		X			X	X	X
6-10	X			X			
11-15			X				
16-20							
21-25							
26-30							
31-35							
36-40							
41-45							
46-50							
51-55							
56-60							
61-65							
66-70							
71-75							
76-80							
81-85							
86-90							
91-95							
96-100							
101-105							
106-110							
111-115							
116-120							
Over 2 hours							

WEEK 32

Minutes	Day 1	Day 2	Day 3	Day 4	Day 5	Day 6	Day 7
0-5					X		
6-10	X	X	X			X	X
11-15				X			
16-20							
21-25							
26-30							
31-35							
36-40							
41-45							
46-50							
51-55							
56-60							
61-65							
66-70							
71-75							
76-80							
81-85							
86-90							
91-95							
96-100							
101-105							
106-110							
111-115							
116-120							
Over 2 hours							

WEEK 33

Minutes	Day 1	Day 2	Day 3	Day 4	Day 5	Day 6	Day 7
0-5							
6-10	X						
11-15		X				X	
16-20			X		X		X
21-25				X			
26-30							
31-35							
36-40							
41-45							
46-50							
51-55							
56-60							
61-65							
66-70							
71-75							
76-80							
81-85							
86-90							
91-95							
96-100							
101-105							
106-110							
111-115							
116-120							
Over 2 hours							

WEEK 34

Minutes	Day 1	Day 2	Day 3	Day 4	Day 5	Day 6	Day 7
0-5							
6-10							
11-15							
16-20							
21-25							
26-30	X						
31-35			X				
36-40		X					
41-45				X	X		
46-50						X	X
51-55							
56-60							
61-65							
66-70							
71-75							
76-80							
81-85							
86-90							
91-95							
96-100							
101-105							
106-110							
111-115							
116-120							
Over 2 hours							

5 minutes per box
